# Optimization of cabbage (*Brassica oleracea* var. *capitata* L.) protoplast transformation for genome editing using CRISPR/Cas9

**DOI:** 10.3389/fpls.2023.1245433

**Published:** 2023-10-02

**Authors:** Ester Stajič, Urban Kunej

**Affiliations:** Department of Agronomy, Biotechnical Faculty, University of Ljubljana, Ljubljana, Slovenia

**Keywords:** CRISPR/Cas9, genome editing, protoplasts, PEG-mediated transformation, B. oleracea

## Abstract

Genome editing techniques, such as Clustered Regularly Interspaced Short Palindromic Repeats/CRISPR-associated systems (CRISPR/Cas9) are undoubtedly becoming an indispensable tool for improving food crops and tackling agricultural challenges. In the present study, key factors affecting transformation efficiency, such as PEG4000 concentration, incubation time, and plasmid amount were evaluated to achieve efficient delivery of CRISPR/Cas9 vector into cabbage protoplasts. Using amplicon sequencing, we confirmed a significant effect of PEG4000 concentration and incubation time on the induced target mutations. By optimizing the transformation protocol, editing efficiency of 26.4% was achieved with 40 µg of plasmid and 15 minutes incubation with 50% PEG4000. While these factors strongly affected the mutation rate, the viability of the transformed protoplasts remained high. Our findings would be useful for successful genome editing in cabbage and other brassicas, as well as in research areas such as gene function analysis and subcellular localization that rely on transient transformation methods in protoplasts.

## 1 Introduction

*Brassica oleracea* includes many important vegetable crops with high nutritional value such as kale, cauliflower, cabbage, broccoli and kohlrabi. Cabbage (*Brassica oleracea* var. *capitata* L.) is an important source of vitamins, folic acid, flavonoids and calcium. In addition, it contains phytochemicals (glucosinolates) that are of great benefit to human health as they have anti-inflammatory and anti-cancer effects (Šamec et al., 2017). Cabbage is a biennial plant with an obligate requirement for vernalization and is self-incompatible (Ma et al., 2019). Therefore, breeding by conventional methods is a lengthy process, and modern biotechnology methods are needed to overcome these limitations.

Doubled haploid (DH) technology by microspore isolation is used in cabbage breeding to shorten the breeding process of elite F1 hybrids. However, regeneration of DH plants is highly dependent on genotype (Rudolf et al., 1999). Haploids could be obtained using haploid inducer lines with mutant alleles that trigger chromosome elimination after pollination, an approach commonly used in maize and other cereals (Ren et al., 2017). One of the genes involved in haploid induction is *CENH3* (centromere-specific histone H3). The role of CENH3 protein in kinetochore formation and the effects of modifications in this protein on chromosome segregation and production of spontaneous haploids after pollination have been described in many publications (Ravi and Chan, 2010; Karimi-Ashtiyani et al., 2015; Kuppu et al., 2015; Kuppu et al., 2020).

Genome editing techniques such as CRISPR/Cas9 can be used to make precise changes in specific regions of the genome. Applications of these methods range from basic biological studies of gene function to agricultural breeding and the development of new plant varieties with improved genomic traits or suppressed undesirable functions (Zhu et al., 2020). *Agrobacterium*-mediated transformations are currently predominant to introduce CRISPR/Cas9 reagents into plant cells (Laforest and Nadakuduti, 2022). However, stable transformation protocols have many limitations as they are time-consuming and labor-intensive. Moreover, to develop an efficient CRISPR/Cas9 protocol, various factors affecting the mutation rate need to be evaluated before practical use. The protoplast-based transient transformation system provides a rapid and efficient platform to validate multiple mutagenesis parameters in a short time. Moreover, protoplasts can be further regenerated into plants (Lin et al., 2018; Nadakuduti et al., 2019). Protoplast isolation and transformation protocols have been reported for numerous agriculturally important crops (He et al., 2016; Xiong et al., 2019; Wang et al., 2021). Some studies have also been published for the genus *Brassica* (Murovec et al., 2018; Stajič et al., 2019; Li et al., 2021; Sivanandhan et al., 2021); however, the effects of PEG-transformation parameters on editing efficiency have not been investigated in detail.

In the present study, we aimed to optimize the conditions for PEG-mediated protoplast transformation to enhance editing efficiency using CRISPR/Cas9 in a non-model horticultural plant by targeting the cabbage *CENH3* gene associated with haploid induction. Amplicon sequencing was used to determine the mutation rate in different transformation experiments. In addition, high viability of transformed protoplasts was confirmed by FDA staining.

## 2 Materials and methods

### 2.1 Plant material

Two cabbage cultivars, namely ‘Rebecca F1’ and ‘Reball F1’ (Syngenta) were used in this study. Seeds were sterilized in a 1.66% (w/v) solution of dichloroisocyanuric acid (Sigma) for 15 min and then rinsed three times with sterile distilled water. Seeds were placed for germination on MS (Murashige and Skoog, 1962) culture medium (MS basal salts plus vitamins, 3% sucrose, 0.8% agar, pH 5.8) in a growth chamber with a 16-hour light/8-hour night photoperiod at 21°C. After one week, seedlings were transferred to ECO2 plastic containers with the same medium and maintained at 21°C until plantlets with developed leaves were obtained.

### 2.2 Plasmid preparation

Single-guide RNAs **(**sgRNAs) targeting the cabbage *CENH3* gene were designed using CRISPR RGEN tools (Park et al., 2015). To clone sgRNAs, primers (**Supplementary Table 1**) were annealed and ligated into pHSN401 (Addgene #50588) using T4 DNA ligase (NEB) and *Bsa*I-HF (NEB) in a Golden Gate reaction. To confirm correct vector construction, we used Sanger sequencing. All vector construction procedures were performed according to Xing et al. (2014).

### 2.3 Protoplast isolation

Protoplasts were isolated according to the protocol of Kiełkowska and Adamus (2012) with minor modifications. Briefly, 1.5 g of young leaves were cut with a sharp razor in a 9-cm Petri dish containing 15 ml of plasmolysis solution (**Table 1**) and incubated for 1 h at 25°C in the dark. After pretreatment, the solution was discarded and 8 ml of enzyme solution was added to the cut leaves. The material was digested overnight at 25°C while gently shaking. The next day, the digested material was filtered through a 40-µm cell strainer and centrifuged at 900 RPM for 5 min. The supernatant was discarded and the protoplast pellet was resuspended in 8 ml of SAH/MES solution and overlaid with 2 ml of W5 solution. After centrifugation at 1,100 RPM for 10 min, the protoplasts from interphase were transferred to a fresh centrifuge tube and rinsed with 10 ml of W5 solution. The protoplast pellet was resuspended in MMG solution at a density of 2.5 × 10^6^ protoplasts/ml. The viability of isolated protoplasts was evaluated by staining with 0.01% fluorescein diacetate (FDA). The percentage of viable protoplasts was calculated based on the number of protoplasts with green fluorescence divided by the total number of cells. Viability assessment was repeated three times.

**Table 1:** Solutions and their composition used for cabbage protoplast isolation and transformation.

Solution name	Composition
Plasmolysis solution	0.5 M mannitol (pH 5.7)
Enzyme solution	0.5% cellulase Onozuka RS (Yakult), 0.1% macerozyme R-10 (Duchefa), 0.4 M mannitol, 3 mM CaCl_2_, 2 mM MES (pH 5.6)
SAH/MES solution	0.5 M sucrose, 1 mM MES (pH 5.7)
W5 solution	154 mM NaCl, 125 mM CaCl_2_, 5 mM KCl, 2 mM MES (pH 5.7)
MMG solution	0.4 M mannitol, 15 mM MgCl_2_, 4 mM MES (pH 5.7)
PEG solution	0.2 M mannitol, 100 mM CaCl_2_, 10-50% PEG4000

### 2.4 PEG-mediated transformation of protoplasts

For protoplast transformation, plasmid (20-80 µg) was added to 200 µl of protoplasts, followed by addition of equal volume of PEG solution. The tubes were mixed gently and incubated in the dark at room temperature for 5 s to 25 min, depending on the protocol tested. After incubation, the reaction was immediately stopped by adding an equal volume of W5 solution followed by two volumes of W5 solution. Samples were then centrifuged at 700 RPM for 5 min, and the supernatant was removed. The transformed protoplasts were washed a final time with 10 ml of W5 solution and resuspended in 1 ml of the same solution. Protoplasts were transferred to 24-well plates (Sigma) and incubated in the dark at room temperature for 48 h. After incubation, protoplast viability was determined as described above, and protoplasts were collected by centrifugation at 12,000 RPM before DNA isolation was performed.

### 2.5 Genomic DNA isolation and Ampliseq

Genomic DNA was extracted from the pellet of protoplasts using E.Z.N.A.^®^ Plant DNA DS Kit (Omega BIO-TEK). Target sites were amplified with Q5^®^ High-Fidelity DNA Polymerase (NEB) using the primers listed in **Supplementary Table 1**. The same target-specific primers followed by unique barcode were used for each sample. PCR was performed with initial denaturation for 30 s at 98°C, 35 cycles of denaturation for 5 s at 98°C, annealing for 10 s at 65°C, elongation for 10 s at 72°C and final elongation for 2 min at 72°C. PCR products were purified using GFX ™ PCR DNA and Gel Band Purification Kit (Cytiva) according to the manufacturer protocol. Samples were then pooled and sequenced on the Illumina HiSeq at Eurofins Genomics Europe Sequencing GmbH (Konstanz, Germany). The obtained next-generation sequencing data were analyzed using Cas-analyzer software (Park et al., 2017) to determine mutation efficiency. Three biological replicates were performed for each experiment and results are presented as the mean ± standard error (SE). Detailed information is provided at the end of the corresponding tables and figures.

## 3 Results

### 3.1 Effect of PEG4000 incubation time on protoplast viability and mutation rate

**Figure 1:**
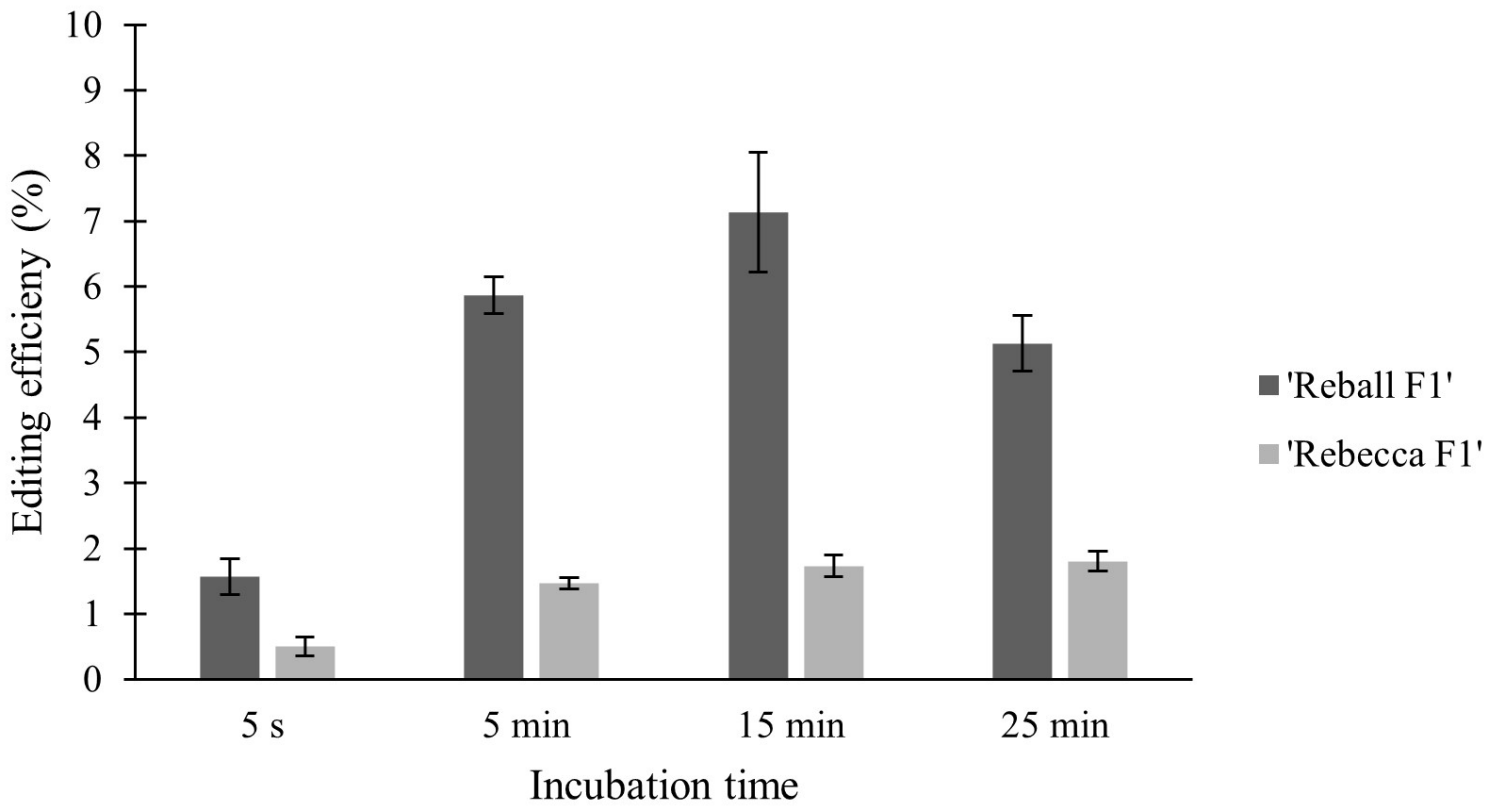
Effect of PEG4000 incubation time on editing efficiency in two cabbage cultivars. Values represent means ± SE of three replicates.

**Figure 2:**
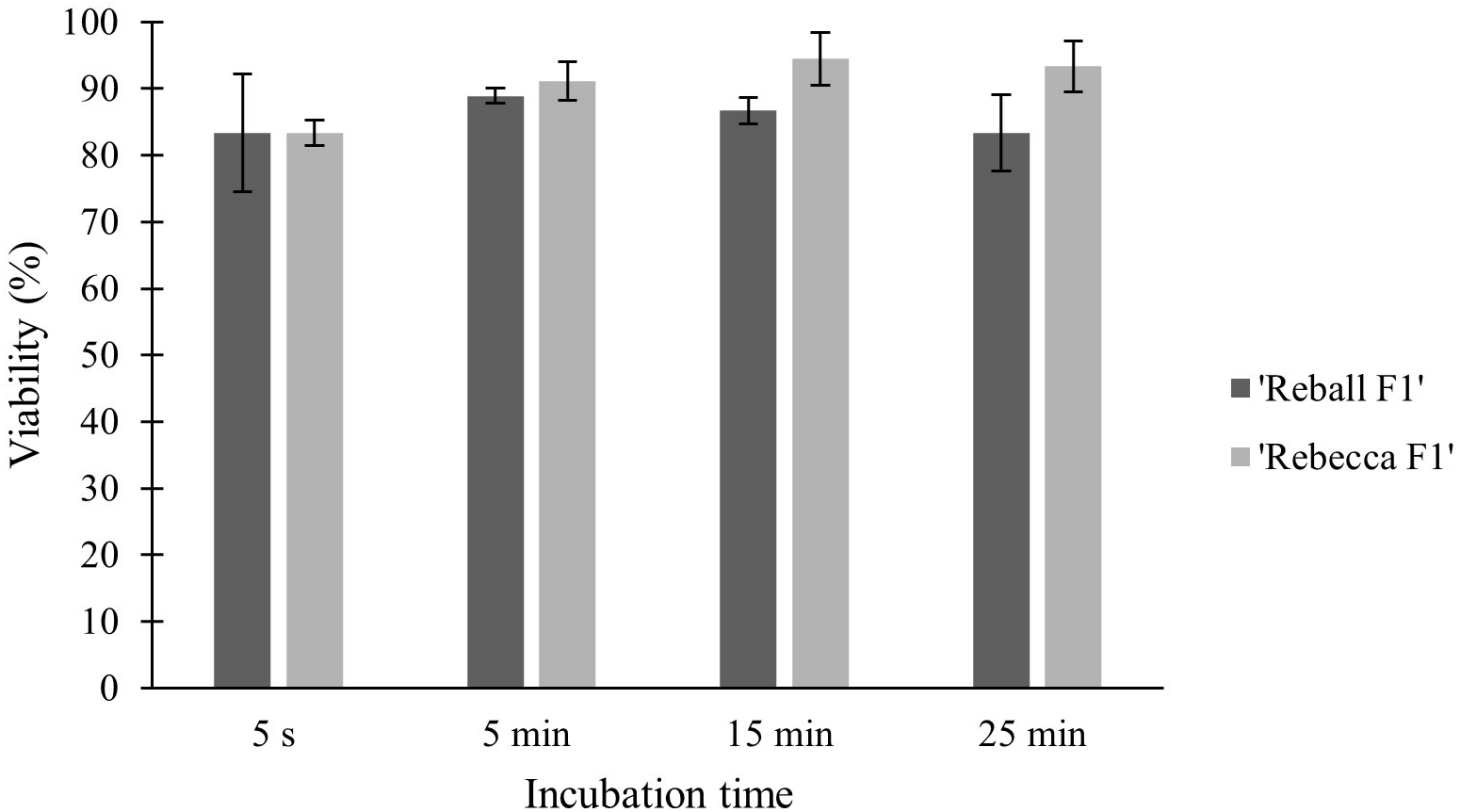
Effect of PEG4000 incubation time on viability of cabbage protoplasts. Values represent means ± SE of three replicates.

High viability and number of protoplasts are one of the main requirements for successful protoplast transformation. Mesophyll protoplasts with high viability (93.6% in cultivar ‘Reball F1’ and 86.7% in cultivar ‘Rebecca F1’) were isolated from young leaves of cabbage plants grown *in vitro* after overnight digestion in 0.5% cellulase Onozuka RS and 0.1% macerozyme R-10. A sucrose density gradient was used to obtain debris-free protoplasts. The average yield of isolated protoplasts was 5.3 × 10^6^ p/g (protoplasts per g of leaves) for the cultivar ‘Reball F1’ and 4.0 × 10^6^ p/g for the cultivar ‘Rebecca F1’.To optimize the PEG-mediated transformation protocol in cabbage protoplasts, the effect of incubation time with PEG4000 on protoplast viability and indel frequencies were investigated. No indel mutations could be detected in control samples transformed without vectors. Editing efficiency in ‘Reball F1’ protoplasts increased from 1.57% to 7.13% within 5 s to 15 min of incubation, but then decreased to 5.13% when protoplasts were incubated with PEG4000 for 25 min. In ‘Rebecca F1’, the detected mutation frequencies were low (0.5-1.8%) regardless of the incubation time (**Figure 1**). Incubation time with PEG4000 did not affect viability, and it remained high (over 83.3% on average) in both genotypes even when the exposure time was extended to 25 min (**Figure 2**).

**Figure 3:**
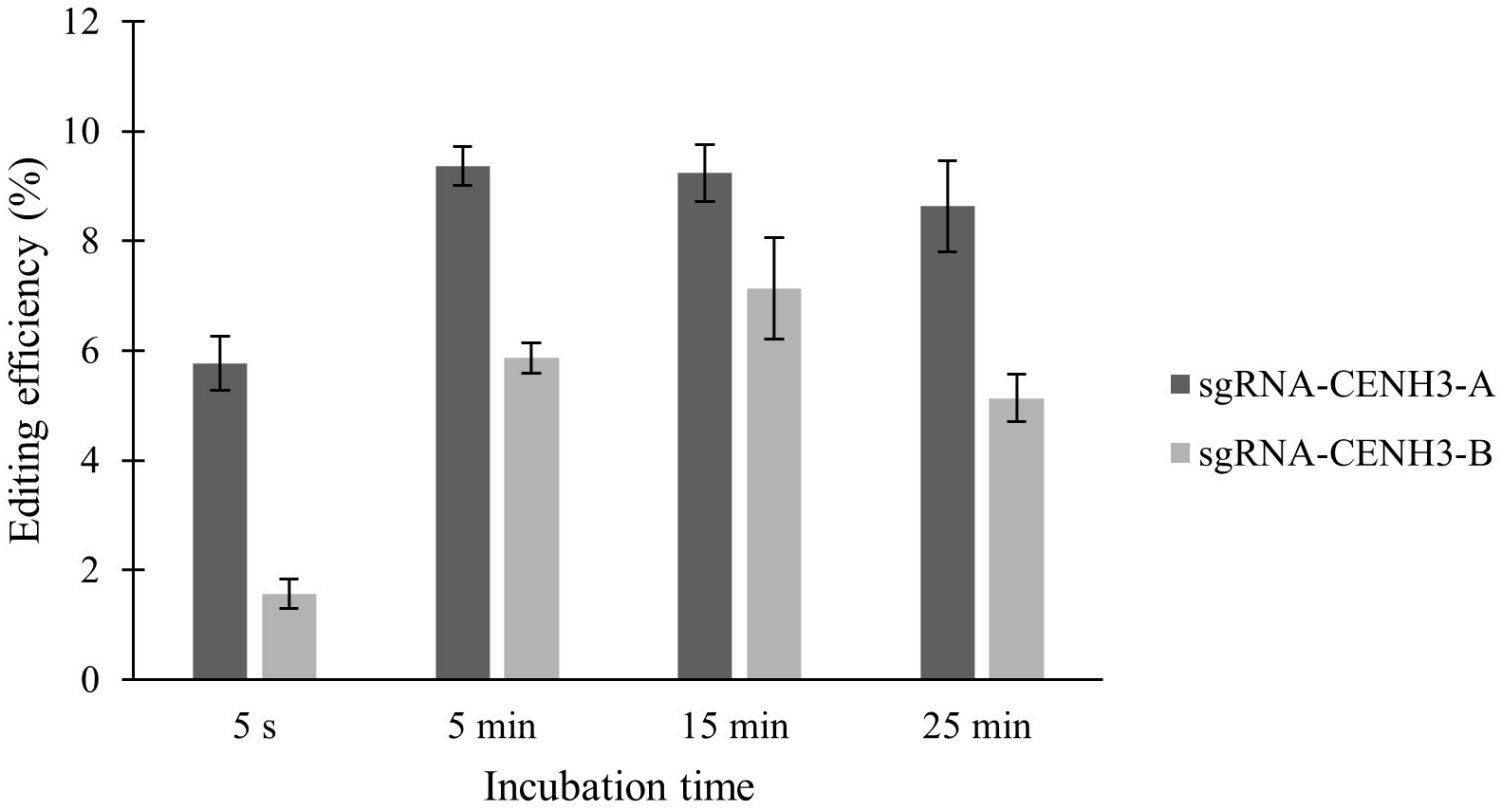
Effect of PEG4000 incubation time on editing efficiency in ‘Reball F1’ protoplasts with two different sgRNAs. Values represent means ± SE of three replicates.

Two sgRNAs targeting the same gene were also tested in ‘Reball F1’ protoplasts. The results showed that higher indel frequencies were obtained with sgRNA-CENH3-A than with sgRNA-CENH3-B (**Figure 3**; **Table 2**). The editing efficiencies ranged from 8.63% to 9.37% when protoplasts were transformed with sgRNA-CENH3-A and the incubation time was 5-25 min. The lowest mutation rate (5.07%) was observed after 5 s incubation with PEG4000.

**Table 2:** Indel frequencies detected in the *CENH3* gene by amplicon sequencing after protoplast transformation with different incubation time.

Genotype	Incubation time	sgRNA	No. of reads analyzed^a^	No. of deletions^a^	No. of insertions^a^	Indel frequency ± SE (%)^a^
‘Reball F1’	5 s	CENH3-A	254,061	8,186	3,656	5.07 ± 0.49
		CENH3-B	35,669	185	311	1.57 ± 0.27
	5 min	CENH3-A	283,542	19,810	6,511	9.37 ± 0.35
		CENH3-B	70,485	1,393	2,723	5.87 ± 0.28
	15 min	CENH3-A	180,306	12,474	4,391	9.23 ± 0.52
		CENH3-B	66,817	1,582	3,009	7.13 ± 0.92
	25 min	CENH3-A	191,875	12,086	4,482	8.63 ± 0.83
		CENH3-B	23,493	487	720	5.13 ± 0.43

a Based on three biological replicates.

### 3.2 Effect of PEG4000 concentration on protoplast viability and mutation rate

**Figure 4:**
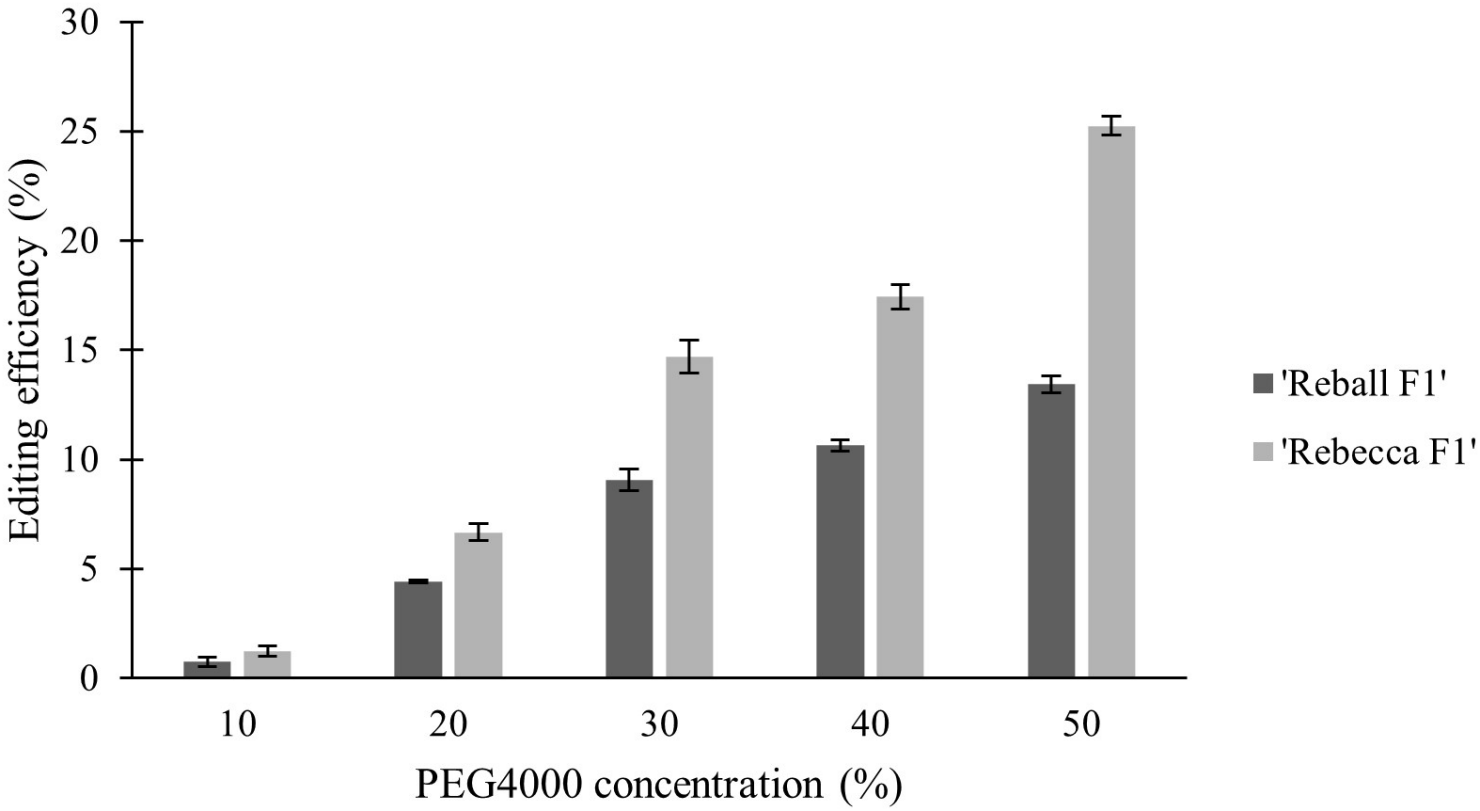
Effect of PEG4000 concentration on editing efficiency in two cabbage cultivars. Values represent means ± SE of three replicates.

**Figure 5:**
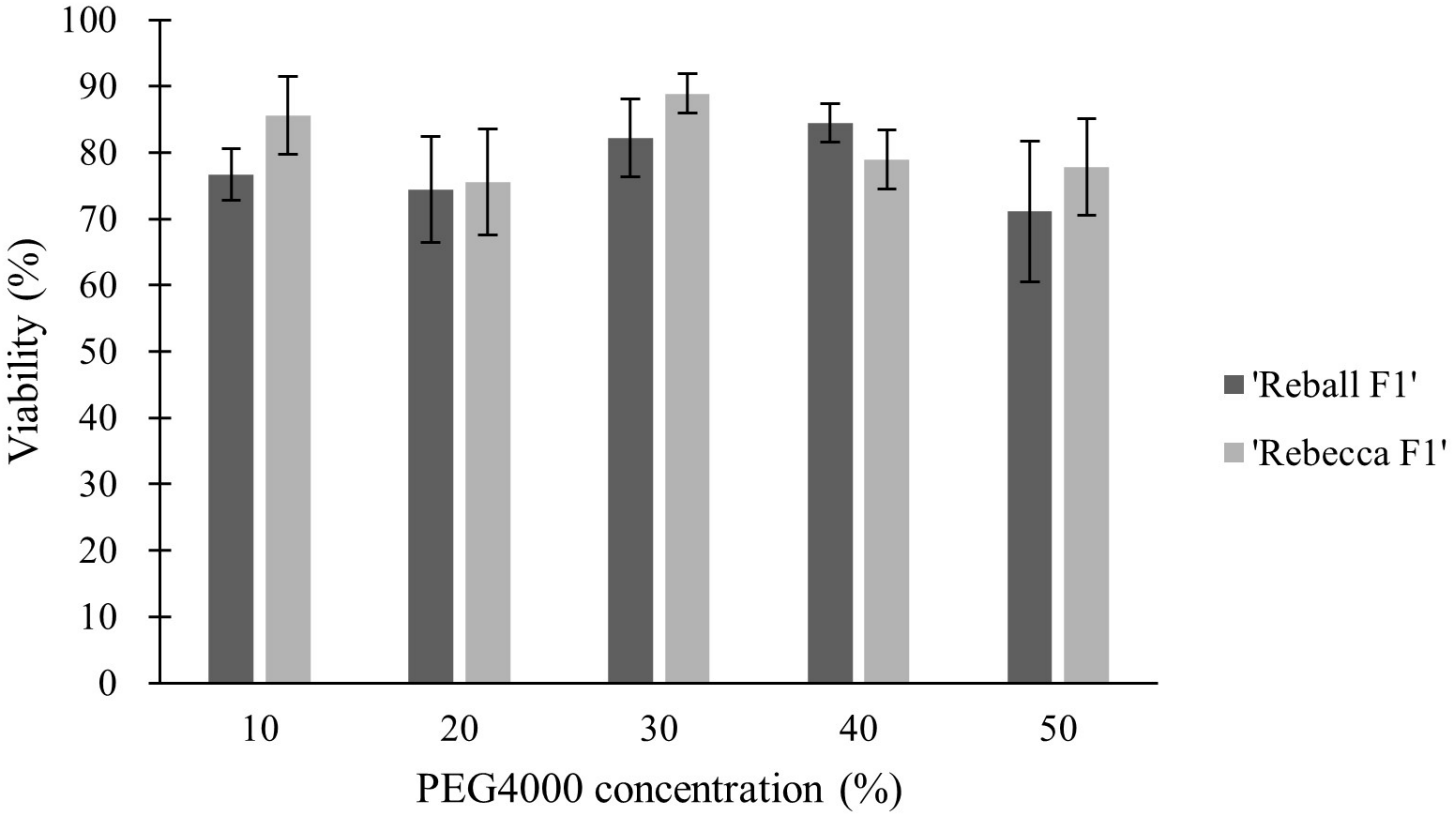
Effect of PEG4000 concentration on viability of cabbage protoplasts. Values represent means ± SE of three replicates.

In the second part of the study, protoplasts of both cultivars were transformed with different concentrations (10-50%) of PEG4000. The PEG4000 concentration substantially affected the efficiency of protoplast transformation. At 10%, the editing efficiency was 0.73% (‘Reball F1’) and 1.23% (‘Rebecca F1’). With increasing PEG4000 concentration, the average mutation frequencies in ‘Reball F1’ and ‘Rebecca F1’ protoplasts increased to 13.43% and 25.23%, respectively (**Figure 4**; **Table 3**). No effect of PEG4000 concentration on the viability of transformed protoplasts of both genotypes was detected (**Figure 5**). Based on these results, 50% PEG4000 was chosen as the optimal PEG4000 concentration.

**Table 3:** Indel frequencies detected by amplicon sequencing after protoplast transformation with different PEG4000 concentrations.

Genotype	PEG4000 concentration (%)	No. of reads analyzed^a^	No. of deletions^a^	No. of insertions^a^	Indel frequency ± SE (%)^a^
‘Reball F1’	10	105,037	613	240	0.73 ± 0.20
	20	119,472	3,895	1,339	4.40 ± 0.06
	30	184,038	12,302	4,473	9.07 ± 0.49
	40	216,983	17,050	6,120	10.63 ± 0.27
	50	78,456	7,691	2,887	13.43 ± 0.38
‘Rebecca F1’	10	165,561	1,598	523	1.23 ± 0.24
	20	181,203	8,851	3,112	6.67 ± 0.37
	30	187,111	19,201	8,165	14.70 ± 0.76
	40	183,655	22,100	9,106	17.43 ± 0.57
	50	67,115	12,003	5,033	25.23 ± 0.43

a Based on three biological replicates.

### 3.3 Effect of plasmid amount on mutation rate and viability

**Figure 6:**
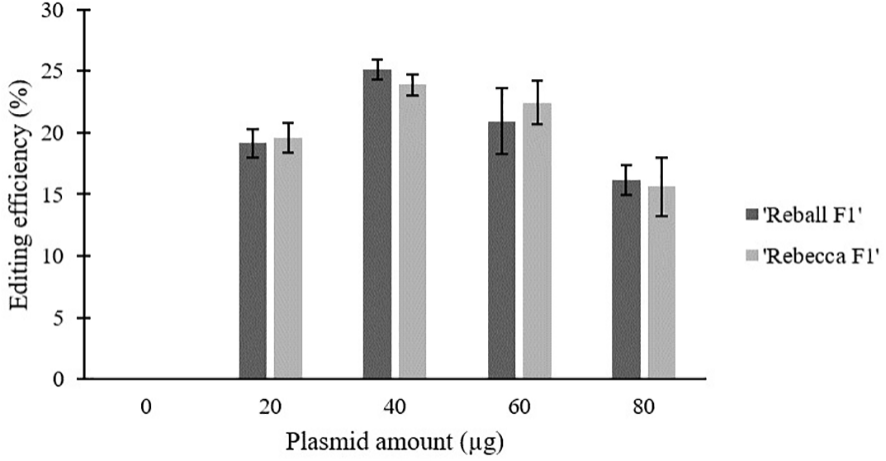
Effect of plasmid amount on editing efficiency in two cabbage cultivars. Values represent means ± SE of three replicates.

**Figure 7:**
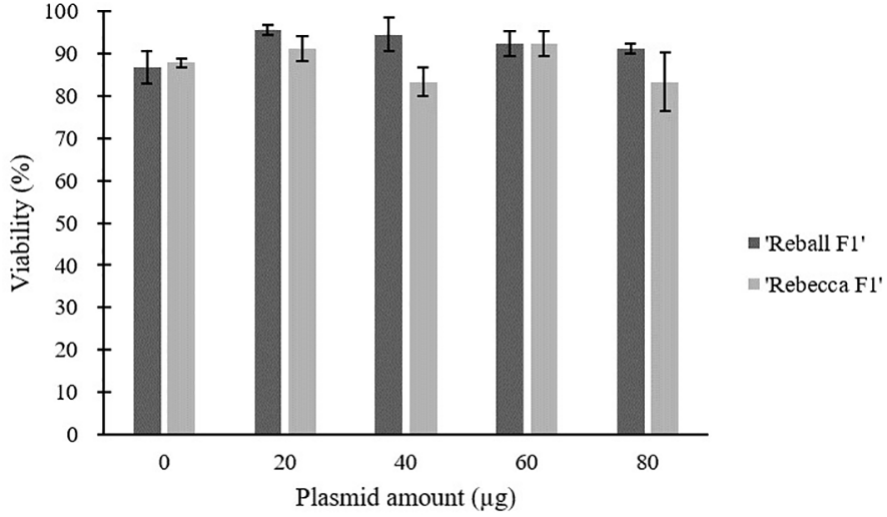
Effect of plasmid amount on viability of cabbage protoplasts. Values represent means ± SE of three replicates.

**Figure 8:**
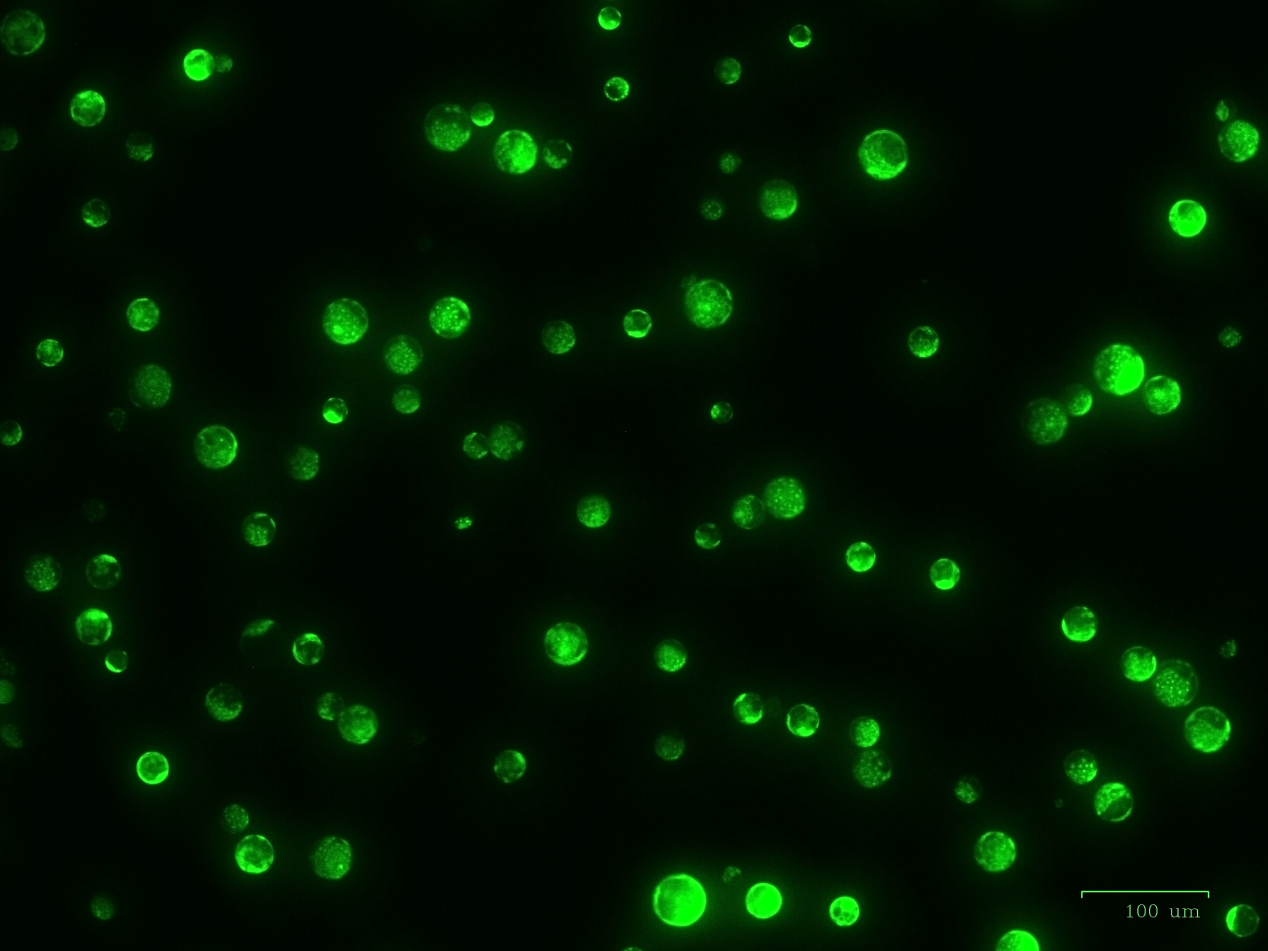
High viability of ‘Reball F1’ protoplasts 48 h after transformation with 80 µg plasmid and 15 min incubation with 50% PEG4000. Scale bar represents 100 µm.

**Figure 9:**
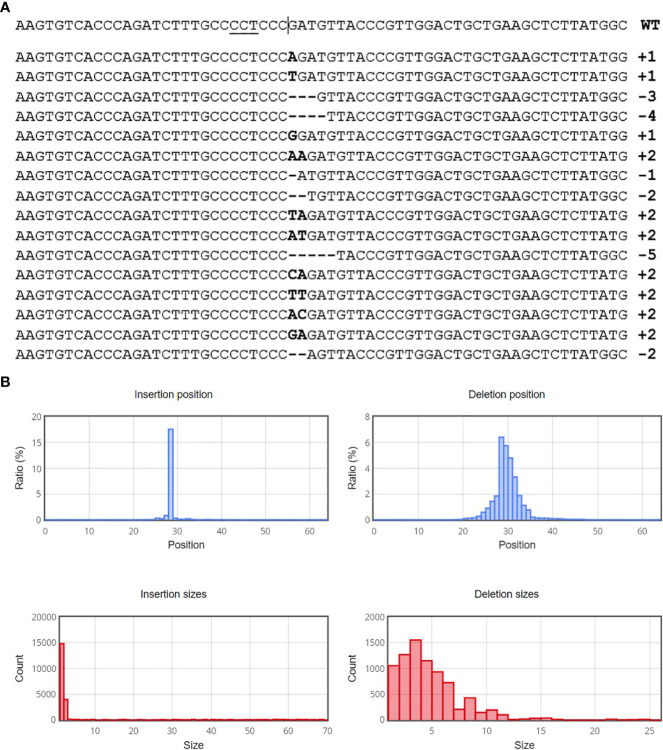
Sequence analysis with Cas-analyzer in ‘Reball F1’protoplasts transformed with 40 µg plasmid, 50% PEG4000 and 15 min incubation. **(A)** Identified alleles around the predicted cleavage site (vertical line) in descending order by read count. Inserted nucleotides are shown in bold font and horizontal dashed lines indicate deletions. **(B)** Size of induced mutations and their distribution.

After optimization of PEG4000 concentration and incubation time, different amounts (20 to 80 µg) of the plasmid were transformed into cabbage protoplasts. The results showed that for both cultivars, mutation rates increased when 40 µg of plasmid was added instead of 20 µg, but then decreased when the amount increased to 60 µg and 80 µg (**Figure 6**; **Table 4**), suggesting that 40 µg of plasmid might be the threshold and was chosen as the optimal plasmid amount for cabbage protoplast transformation. However, viability remained high (91.1% for ‘Reball F1’ and 83.3% for ‘Rebecca F1’) even when 80 µg of plasmid was used for transformation (**Figures 7**, **8**). **Figure 9** shows the most frequent alleles found around the cleavage site in the protoplast sample of ‘Reball F1’ transformed with 40 µg plasmid, 50% PEG4000, and 15 min incubation. The most frequent mutations were 1 bp insertions, followed by 3 bp deletions.

**Table 4:** Indel frequencies detected in the *CENH3* gene by amplicon sequencing after protoplast transformation with different amounts of plasmid.

Genotype	Plasmid amount (µg)	No. of reads analyzed^a^	No. of deletions^a^	No. of insertions^a^	Indel frequency ± SE (%)^a^
‘Reball F1’	0	179,802	0	5	0.00 ± 0.00
	20	140,051	19,935	7,425	19.13 ± 1.19
	40	188,124	33,954	13,742	25.13 ± 0.78
	60	113,868	16,442	7,131	20.93 ± 2.70
	80	145,988	17,217	6,570	16.20 ± 1.21
‘Rebecca F1’	0	166,441	0	3	0.00 ± 0.00
	20	115,687	15,992	6,781	19.57 ± 1.19
	40	264,442	42,655	20,897	23.87 ± 0.87
	60	94,288	15,067	6,472	22.43 ± 1.76
	80	75,695	8,233	3,584	15.63 ± 2.38

a Based on three biological replicates.

## 4 Discussion

The rapidly growing world population and unpredictable environmental changes pose challenges to agricultural production that need to be addressed. Modern genetic tools such as CRISPR/Cas9 provide the opportunity to introduce precise genetic changes and develop new varieties with higher yields, better quality, and improved resistance (Zhu et al., 2020). However, a successful genome editing protocol depends on a combination of factors, and efficient delivery of genome editing reagents into plant cells remains the major bottleneck in plant genome editing (Laforest and Nadakuduti, 2022). Currently, protoplast transformation is gaining interest because it can be used in various areas of plant science research and protoplasts can also be regenerated into modified plants without the incorporation of foreign DNA (Nadakuduti et al., 2019; Park et al., 2019). For protoplast transformation and further regeneration into plants, yield, viability, and transformation efficiency are important factors.

In our experiments, protoplasts from two cabbage cultivars were isolated with high yield and viability, which indicates that our protoplast isolation protocol is suitable to obtain high-quality protoplasts appropriate for downstream applications. To optimize the PEG-mediated delivery of CRISPR/Cas9 plasmids, we targeted the *CENH3* gene of high breeding importance. Editing this gene could lead to the development of a haploid inducer line and the generation of haploids in recalcitrant genotypes (Kuppu et al., 2020). The efficiency of CRISPR/Cas9-induced mutations in protoplasts varies greatly, even among *Brassica* species. Murovec et al. (2018) detected only 2.25% of indel mutations in cabbage protoplasts when RNPs were introduced, whereas mutation rates were higher in *Brassica rapa* (up to 24.51%). In our previous report, editing efficiencies of 1.27-11.95% were obtained in cabbage protoplasts after plasmid transformation (Stajič et al., 2019). We therefore decided to test different transformation parameters (PEG4000 concentration, incubation time, and plasmid amount) to achieve higher editing efficiencies.

Our data suggest that prolonged incubation of 15 to 25 min with PEG4000 leads to a decrease in editing efficiency, which was confirmed with two different sgRNAs. In contrast, Sivanandhan et al. (2021) found that incubation for 30 min instead of 20 min positively affected transformation efficiency in *Brassica rapa* protoplasts. In most protocols, 15 min of exposure time and 40% PEG4000 are taken as standard. PEG4000 concentration substantially influenced editing efficiency in our experiments and increased significantly when PEG4000 concentration was increased to 50%. For broccoli protoplasts, 20% PEG4000 was determined as the optimal concentration, as higher concentrations led to protoplast rupture (Yang et al., 2022). Similarly, a concentration of 25% PEG4000 was used for the transformation of *Brassica napus* protoplasts (Li et al., 2021). In our experiments, the viability of transformed protoplasts remained high even when 50% PEG4000 was used. Another important factor influencing transformation efficiency in protoplasts is amount of plasmid. Optimal dosage of plasmid differs greatly, in broccoli protoplasts, no differences in transformation efficiency were found when 5-15 µg of plasmid was used (Yang et al., 2022), while 40 µg of the plasmid was reported as optimal amount in *Brassica rapa* protoplasts (Sivanandhan et al., 2021). Transformation of cabbage protoplasts with 40 instead 20 µg of plasmid improved the editing efficiency in our experiments. However, higher amounts (60 and 80 µg) had a negative effect.

## 5 Conclusion

The results obtained in this study show that higher editing efficiency can be achieved by refining the transformation protocol. The data collected in our experiments show not only high viability of isolated protoplasts but also higher success than other current protocols in *Brassica oleracea*, with editing efficiency reaching 26.4% and 25.4% in ‘Reball F1’ and ‘Rebecca F1’ protoplasts, respectively, by subjecting protoplasts to optimized conditions (15 min incubation with 50% PEG4000 and 40 μg plasmid), while remaining high viability. All these data indicate that this isolation and transformation protocol is highly efficient and could be further used for plant regeneration from protoplast to obtain modified plants. Our optimized protocol could also be used for further optimization of editing efficiency (e.g. enhancing expression of CRISPR/Cas9 reagents) and would contribute to successful genome editing in other brassicas as well.

## Data availability statement

The original contributions presented in the study are included in the article/**Supplementary Material**. Further inquiries can be directed to the corresponding author.

## Author contributions

ES conceived the project. UK contributed to data analysis. ES wrote the first draft of the manuscript. Both authors performed the experiments and contributed to the manuscript revision. All authors contributed to the article and approved the submitted version.
