# Supplementary material for: Optimization of cabbage (Brassica oleracea var. capitata L.) protoplast transformation for genome editing using CRISPR/Cas9
Source: Front Plant Sci. 2023 Oct 2;14:1245433. doi: 10.3389/fpls.2023.1245433 (PMC10577288; doi:10.3389/fpls.2023.1245433)
Supplement: Supplementary file 1 [file Table_1.docx]

| **Primer Name** | **Sequence (5ˈ- 3ˈ)** | **Purpose** |
| --- | --- | --- |
| sgRNA-CENH3-A-For | attgGTCCAACGGGTAACATCGGG | CRISPR/Cas9 construct preparation |
| sgRNA-CENH3-A-Rev | aaacCCCGATGTTACCCGTTGGAC |  |
| sgRNA-CENH3-B-For | attgGCACGCCGTCTTGGAGGAAA |  |
| sgRNA-CENH3-B-Rev | aaacTTTCCTCCAAGACGGCGTGC |  |
| NGS-CENH3-A-For | AAGTGTCACCCAGATCTTTGC | Amplicon sequencing |
| NGS-CENH3-A-Rev | TCAGAGAACAAGCCAATTAAAAA |  |
| NGS-CENH3-B-For | GATGCTATGCTTTGCGCTATC |  |
| NGS-CENH3-B-Rev | TACAACCTGCGAAACGATCA |  |

**Table S1:** Details of primers used in the study
